# Interrupted Aortic Arch in a Patient with Patient-Prosthesis Mismatch after Aortic Valve Replacement

**DOI:** 10.1055/s-0039-3401010

**Published:** 2020-02-04

**Authors:** Ziv Beckerman, LaRonica McPherson, Edward P. Chen

**Affiliations:** 1 Department of Surgery and Perioperative Care, University of Texas Dell Medical School, Austin, Texas; 2 Texas Center for Pediatric and Congenital Heart Disease, University of Texas Health Austin/Dell Children's Medical Center, Austin, Texas; 3 Division of Cardiothoracic Surgery, Department of Surgery, Emory University School of Medicine, Atlanta, Georgia

**Keywords:** patient-prosthesis mismatch, interrupted aortic arch, Ross procedure, aortic stenosis, mitral insufficiency

## Abstract

This case presents a patient who underwent aortic valve replacement and presented 13 years later with high gradients across the prosthesis, mitral insufficiency, and severe systemic hypertension. Her preoperative workup led to the diagnosis of an interrupted aortic arch Type A. Her surgical management included an initial procedure to repair the interruption, and 11 months later after resolution of her hypertension, a second surgery, which included the Ross procedure and mitral valve repair.

## Introduction

Interrupted aortic arch (IAA) is a rare and severe anomaly, accounting for approximately 1% of congenital cardiovascular defects. It can be subdivided into the following three groups with respect to the site of interruption: Type A, interruption distal to the left subclavian artery; Type B, between the left carotid and left subclavian arteries, and Type C, between the innominate and left carotid arteries. The most common form in newborns is Type B (70–75%), the least common form is Type C (2–4%). 1

IAA Type A and severe cases of aortic coarctation may show close anatomical and clinical resemblance. Aortic coarctation occurs far more frequently and accounts for 8% congenital heart defects. Coarctation of the aorta is thought to occur by contraction and fibrosis of anomalous circumferential fibroductal tissue that pulls the posterior aortic shelf toward the contralateral wall. This process, in its most severe form, could result in IAA. 2

Without surgical intervention, 76% of the patients with IAA will die in the newborn period and 90% in the first year of life. 3 The natural history of isolated IAA and coarctation is also similar among adult survivors, who typically present late onset of symptoms and a well-developed collateral circulation bypassing the obstruction. 4

There is a predominance of Type-A IAA in adults, presumably because of the progression of severe coarctation to complete occlusion. 5 Hypertension refractory to medical management is seen in 70% of these patients. Other symptoms include claudication (13%), aortic insufficiency (AI; 10%), and congestive heart failure (6%).

## Case Presentation

A 41-year-old female with a past medical history of bicuspid aortic valve (BAV), s/p aortic valve replacement (AVR; 21-mm St. Jude mechanical prosthesis) due to severe AI at 28 years of age, and severe systemic hypertension was referred to our institution for worsening dyspnea and congestive heart failure. Following her index operation, performed at an outside institution, the patient had an uneventful postoperative course and was discharged home on postoperative day (POD) 5. The patient thereafter continued visiting her primary provider for treatment of hypertension (which required three pharmacological agents).

Upon referral, transthoracic echocardiography (TTE) demonstrated moderate-to-severe mitral regurgitation (MR) and moderate–aortic valve stenosis. The patient was presented with several surgical options to address her cardiac pathology; she elected to undergo the Ross procedure to address her aortic valve and eliminate anticoagulation, with an additional mitral valve repair.

**Fig. 1:**
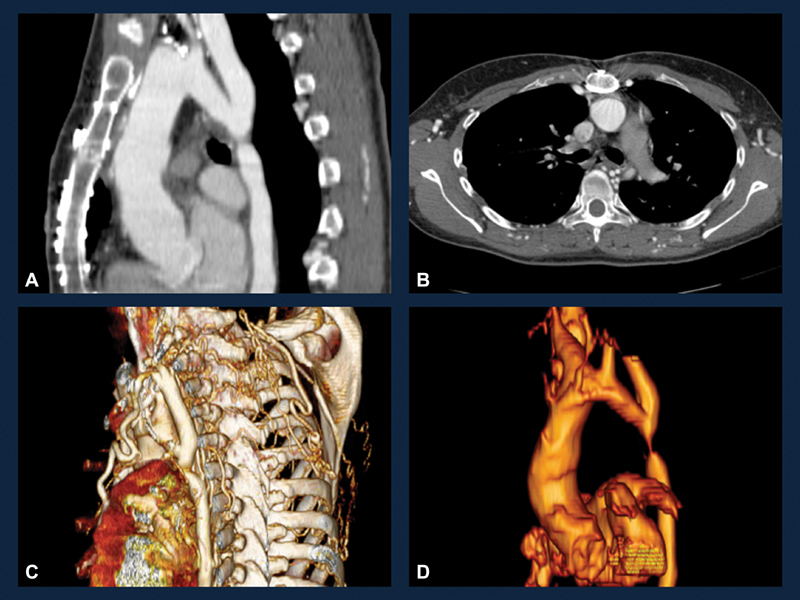
Computed tomography angiography of the chest demonstrating interrupted aortic arch. ( **A** ) Sagittal section of the chest; ( **B** ) cross sectional image showing multiple collateral vessels in the place of the descending aorta location; ( **C** ) 3D reconstruction of the chest demonstrating the severe coarctation-near interruption and numerous collateral vessels); and ( **D** ) 3D reconstruction of the aorta.

Preoperative evaluation was undertaken. TTE demonstrated normal biventricular ejection fraction, aortic valve prosthesis with peak pressure gradient (PPG) of 31 mm Hg, and mean pressure gradient (MPG) of 18 mm Hg, as well as a myxomatous mitral valve with moderate-to-severe MR. Cardiac catheterization via the right radial artery demonstrated normal coronary anatomy. Preoperative computed tomography angiography (CTA) of chest revealed a focal narrowing and near interruption of the proximal descending thoracic aorta just distal to the origin of the left subclavian artery ( Fig. 1 ). Numerous collateral vessels were seen within the posterior mediastinum and chest.

Accordingly, the diagnosis of an aortic interruption/obstructed aortic coarctation was made. A decision was made to address the aortic pathology first via the left chest, allowing for postoperative recovery and potential improvement in hypertension.

**Fig. 2:**
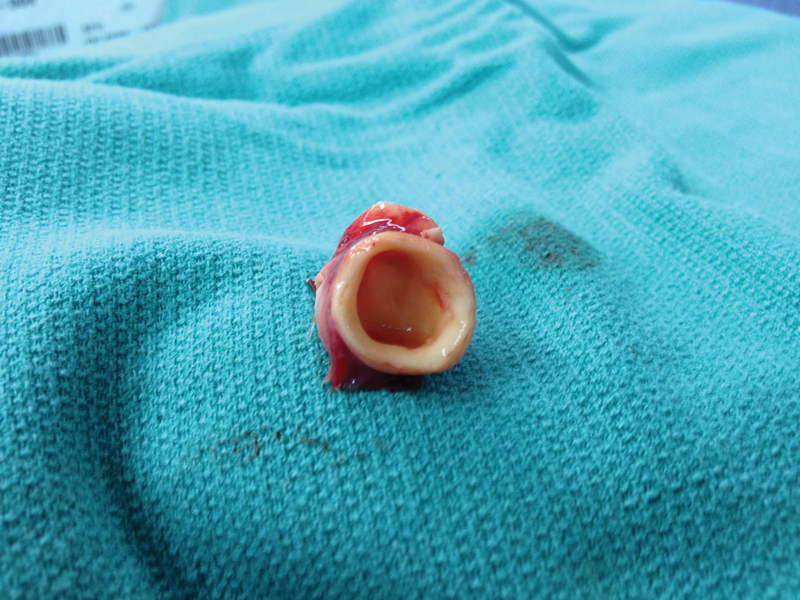
Surgical specimen of the descending aorta.

After appropriate preparation, the patient was taken to the operating room. There was a 35 to 40 mm Hg difference in the blood pressure between the upper and lower extremities. A major left thoracotomy incision was made, and the chest was accessed via the fourth intercostal space. The aorta was mobilized, and the narrowed segment was identified just beyond the left subclavian artery. Under left heart bypass, the subclavian artery and proximal arch were clamped. The coarctation-interrupted arch segment was completely excised as an en bloc specimen. Inspection of the specimen showed absolutely no lumen at all through this narrowed area ( Fig. 2 ).

A 14-mm Gelweave graft was used for descending thoracic aortic replacement. The blood pressures in the femoral and radial arteries at the end of the case were completely equal. Her postoperative course was unremarkable, and she was discharged home on POD 7.

The patient recovered nicely from the surgery, her hypertension was essentially resolved, and she was weaned from all antihypertension medications. A 6-month follow-up CT scan showed no further evidence of aortic arch narrowing. However, she continued to have shortness of breath and dyspnea on exertion. Repeat echocardiography demonstrated preserved ventricular function. The gradient across the aortic valve had a mean of 19 mm Hg, with moderate-to-severe mitral insufficiency. Given the residual symptoms and the patient's wish to stop taking anticoagulation, it was decided to proceed with cardiac repair.

Her second operation took place 11 months after the previous surgery through the previous sternotomy. After transecting the aorta, the valve was inspected. It seemed to be working appropriately but there was a significant amount of pannus overgrowing the sewing ring of the valve. The Ross procedure was performed, and the mitral valve was repaired. The patient's postoperative course was unremarkable, and she was discharged home on POD 5.

**Fig. 3:**
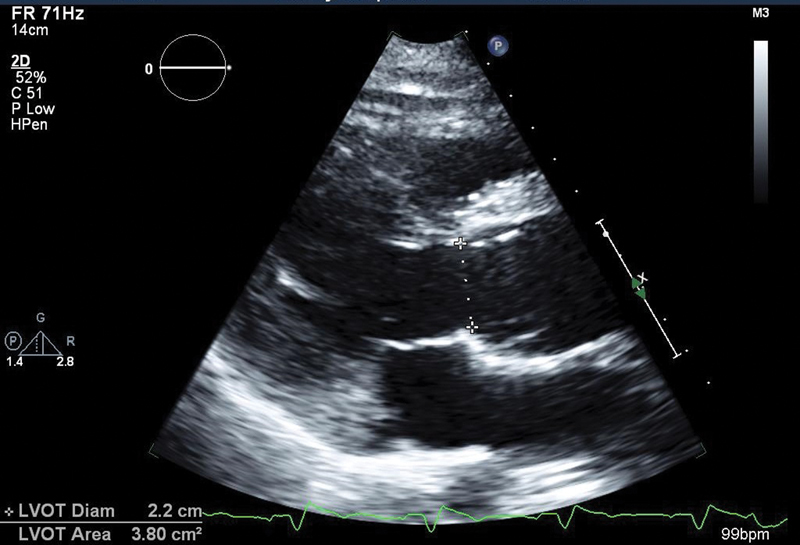
Echocardiogram after the Ross procedure, aortic root.

At her last visit (3 years after surgery), she reported relief of all dyspnea and her subjective quality of life was significantly improved. TTE demonstrated preserved biventricular function, trivial AI, trivial PI, and minimal MR ( Fig. 3 ).

## Discussion

To our knowledge, this is the first case in the literature of a patient presenting with an IAA which was not diagnosed in a patient who previously underwent AVR due to BAV with severe AI. Although IAA can be fatal when presenting during infancy, patients without shunt lesions may survive to adulthood with adequate development of collateral blood supply.

The preoperative workup of this patient stresses the clinical importance of a preoperative CTA. Several surgical options existed for managing the cardiac and aortic findings in this patient. Due to the lack of luminal continuity of the aorta, catheter-based interventions were not believed to be an option.

We elected not to repair the aorta and the heart at the same setting, mainly because of the patient's wish to undergo the Ross procedure. Performing the Ross procedure in a patient with severe systemic hypertension may entail poor prognosis and outcome for the autograft. Thus, the decision was made to perform the repair in two sessions, allowing for potential resolution of the systemic hypertension after the IAA repair, and reassessment of the clinical and echocardiographic findings after reducing the afterload to the heart.

The authors conclude that (1) severe systemic hypertension in a young patient with history of BAV should raise the suspicion of an aortic coarctation and/or IAA, (2) CTA of chest should be a routine prior to repeat sternotomies for cardiac repair, and (3) if the Ross procedure is considered in such a patient, repair of the aortic pathology should be considered first to provide the ideal prognosis for the autograft.

## Notes

Editor's Commentary 1. You emphasize the importance of performing a pre-operative computed tomography (CT) scan. However, should not this aortic interruption have been easily detected on physical exam? The difference in pulses between the upper and lower extremities must have been dramatic before both the initial surgery and the subsequent aortic arch repair.
*I completely agree that, though with a significant collateral burden, the actual pulse propagation was able to be sensed and the patient did have weakened but palpable pulses in lower extremities. I believe that in part, that led to missing the diagnosis before the first operation.*
2. Do you ever resect the pannus from mechanical valves without changing the valve?
*I have done that in the past, mostly as a bailout procedure. For e.g.example, the last case I can recall was a young man, 4fourth time redo that needed replacement of his mechanical aortic valve for PPM , redo of his Konno's aortoplasty and his mechanical mitral valve had a 12 mm Hg mean gradient across. We ended up doing a redo Konno, Bentall root replacement, and removing the pannus from the mechanical mitral instead of replacing it, due to an already prolonged cross-clamp time and a MV which seemed to be restricted from the pannus only.*

## References

[1] Schreiber C Eicken A Vogt M Repair of interrupted aortic arch: results after more than 20 years Ann Thorac Surg 2000 70 06 1896 1899, discussion 1899–1900 11156091 10.1016/s0003-4975(00)01858-0

[2] Ho S Y Baker E J Rigby M L Anderson R H Aortic coarctation and interruption. Color Atlas of Congenital Heart Disease. Morphologic and Clinical Correlations London, UK Mosby-Wolfe 1995 171 178

[3] Schumacher G Schreiber R Meisner H Lorenz H P Sebening F Bühlmeyer K Interrupted aortic arch: natural history and operative results Pediatr Cardiol 1986 7 02 89 93 3797292 10.1007/BF02328957

[4] Dische M R Tsai M Baltaxe H A Solitary interruption of the arch of the aorta. Clinicopathologic review of eight cases Am J Cardiol 1975 35 02 271 277 1119388 10.1016/0002-9149(75)90012-0

[5] Gordon E A Person T Kavarana M Ikonomidis J S Interrupted aortic arch in the adult J Card Surg 2011 26 04 405 409 21793929 10.1111/j.1540-8191.2011.01273.x

